# Of the Experimental Method in Physiology—of the Sensitive and Motor Nerves, and the Ideas of Sir C. Bell

**Published:** 1839-06-08

**Authors:** 


### *Of the Experimental Method in Physiology—Of the Sensitive and Motor Nerves, and the Ideas of Sir C. Bell.* (*Discourse delivered at the Academy, by Professor* GERDY,* *during the seance of 16th April.*)

[Translated for the Medical Examiner, from L'Expérience, Journal de Médecine et de Chirurgie.]

I regret to find myself still disagreeing with colleagues so distinguished as my honourable opponents. But having been myself seriously engaged for fifteen or twenty years in researches in phy-

* This discourse has been taken down with the greatest care, during the sitting of 16th April, written out under the eyes of M. Gerdy, and revised by him. These, then, are his opinions, which we here reproduce in all their integrity.

siology, I ought to be expected to make in it a certain number of new observations, and arrive by my own experience, at definite ideas of the relative value of the different methods of study proper to an advancement of the science. You already foresee that if I do not look upon vivisections as the only good method, it is because I have received very great advantages from another, and have been deceived in my hopes with regard to vivisections. Well, gentlemen, this is precisely what has happened. On the one hand, in my courses on experimental physiology, having several times announced the results, I have sometimes had the displeasure (and shall I say, the shame?) to see happen, precisely the reverse of what I had predicted; at other times the results were so obscure, that it was impossible to find in them the proof that I sought, and which was promised. These public failures, and many others which I experienced in my private researches, are the circumstances which have shaken my faith in the power of vivisections. On the other hand, I have experienced such great advantages from other methods of studying physiology, or, if you please, from a *complex*, *analytical*, and *logical* method, to which I owe so large a number of new observations, that I ought to grant it great confidence and esteem, and much more than they do to it in the experimental school.

Consequently, after having fixed your attention on the functions and faculties of the nerves, I shall entertain you, very rapidly, if you will indulge me, with the success of the complex method of which I come to speak.

But, beforehand, I desire to reply to the objections of my honourable colleague, M. Rochoux, whilst they are yet recent, and to pay to MM. Blandin and Bouillaud a small arrear which I owe them. M. Rochoux has so early set out with such elevated philosophical considerations, that it was very difficult for him not to descend, in proceeding in the discussion. This is what has happened, and he has not thereby become more positive.

In order to justify the system of Sir C. Bell, he has fortified himself with the experiments of which M. Nonat spoke at one of the last sittings. Finding the results of the section of nervous roots upon dogs and rabbits a little obscure, this young physician has practised them on frogs, and he pretends that the results are more evident. How can M. Rochoux invoke similar experiments! What! the inferior animals, like insects, offer such great differences in the functions and properties of the nervous system, that decapitation does not hinder them from walking, running, and sometimes even from flying; salamanders and turtles live whole months with their natural sensibility and agility, notwithstanding the removal of almost the whole head; and do you think it possible to apply, with certainty, to man, experimental observations made on animals, which are so distant from him in their organization and faculties! Besides, do you not perceive that the experiments which you cite with so much confidence, turn against you? In short, if the experiments of M. Magendie on the section of nervous roots, upon the dog and the rabbit, prove the doctrine of Sir C. Bell, why seek among frogs, proofs which lose as much more of their weight, as these animals are lower in the zoological scale?

According to M. Rochoux, the experimental method is the only one which can in the sciences lead to certain results, for it is founded on *experience*, and he brings forward his knowledge of the observations furnished by time; in short, in accordance with the opinion of M. Rochoux, the concessions which I have made with regard to it are insufficient.

Our colleague here plays upon words; he confounds voluntarily *experience*, which is the science of the past, the science of that which we have seen, with *experiment*, which is an operation by which we force nature to speak when we are ready to hear her. Have I not already explained myself several times on this subject? Have I not said that to *observe* is to listen to nature when she herself spontaneously speaks; that to *experiment*, is to listen to her responses when she has been interrogated; and that if by *experience* we understand all knowledge acquired by simple observation, and by experimental observation, it is not what we generally understand by the experimental method? Have I not already remarked, that we should not confound observers in medicine, properly so called, with experimenters? Have I not said here several times, that what I particularly condemn in the experimental method in physiology, is the unlimited confidence and superiority which it grants to vivisections, over other methods of study? Why, then, equivocate in terms, to invest one with paradoxical opinions, and give himself an appearance of reason? I have made no concessions, nor have I any to make; and, in proof of it, all the observations which I shall make to-day, were expressed by me in 1821, in the Supplementary Journal of Medical Sciences, and in 1831, in the Introduction to my Physiology.

Let us pass, then, to the objections of M. Blandin.

"The roots of the spinal nerves are," says he, "proportioned in their volume to the sensibility and movements of the parts, to which they are distributed." But this proposition, generalized into a law, supposes observations made on a great number of animals; and, if I mistake not, M. Blandin has, with a few exceptions, spoken only of those observations made on dogs. This assertion yet implies that we know perfectly well the sensibility and the movements of the different parts to which the nerves distribute themselves. Now, it is precisely the contrary which is true, for we know nothing positive in this respect, and it would even be very difficult to arrive at anything very certain.

The aid of comparative anatomy and physiology has too often been improperly invoked, to illustrate the faculties and the ill-understood functions of the human species. They do not observe that zoological differences, that is to say, differences of organization among animals, necessarily involve differences of faculties and functions. For instance, because it has been demonstrated by M. Edwards that the skin subserves much to respiration with the frog, does it prove that it should do so with us? Because we smell by the nose, does it prove that fishes do the same? If they recognise odours by the aid of their nostrils, it is assuredly by another mechanism that we recognise them. With us, odours make no impression, if we do not breathe through the nose. Plunged in a scented atmosphere, we perceive the odour, when in respiring the air passes through the nostrils. Well, the nostrils of fishes forming two cavities which neither open into the mouth nor into the fauces, but two blind cavities or culs-de-sac, the water does not traverse them during the respiration of the animal.

Lastly, a well-known fact will render my position still more evident. Many authors have asserted that the external ear serves to collect sounds, and to augment their action on the auditory nerve; and they adduce, to prove it, the perfect acoustic trumpets formed by the ear of the horse, of the ass, and of other animals. We now know very positively that the external ear favours audition very little, and that it is not in collecting sounds which assist it. The experiments of M. Lavart have shown that it is by other mechanism.

According to M. Blandin, there is not only an analogy, but a similitude, (that is to say, more than an analogy,) between the nerve of the fifth pair and the vertebral nerves; for the trigeminus has two orders of roots, and that of sensation has a ganglion, as is observed in the spinal nerves. This is what M. Blandin quotes from Sir C. Bell, who found it necessary to establish these analogies for his system. But Gall, who also had a system to establish on the origin of the nerves, has seen these things in a different light.

In reality, gentlemen, see what wide differences exist between the trigeminus and the spinal nerves! The latter have anterior and posterior roots, the trigeminus has them superficial and deep; the anterior roots of the spinal nerves end in the anterior column, and the posterior ones in the posterior column, without our being able to detect their disappearance in the spinal marrow; the superficial roots of the trigeminus, which, for the most part, make the fasciculus called muscular, do not come from the spinal marrow. They form one or more fasciculi, which, leaving the nerve which they help to compose, lose themselves at the surface of the pons varolii, above, below, or within the middle of the crus cerebelli, and disappear sooner or later in different subjects. The deep ones bury themselves among the transverse and longitudinal fibres of the pons in the cineritious and reddish-gray substance which is there observed, and make up at least two large roots—one transverse, the other vertical. The first comes from the inferior extremity of the aqueduct of Sylvius, from the cineritious substance which covers it, in such a manner that it is connected with its corresponding nerve of the opposite side, by this gray matter. The deep vertical root is traced below into the thickness of the restiform body, or between this and the corpus olivare. Now, since the corpus restiforme is the termination of the posterior column of the medulla, the trigeminus has connection with only one of the columns, and it still has other connections with the tissues and adjacent organs which the pons offers in its organization, compared with that of the spinal marrow.

It follows from this arrangement that the fifth pair has an evident connection with the spinal cord only by its deep root, which is traced into the posterior column; that it is not manifestly continuous with the anterior columns, or with the fibres of the corpora pyramidalia; that it only approaches and is contiguous in passing by their side; that, besides, the superficial roots lose themselves immediately at the surface, or in the superficial transverse fibres of the pons varolii, which go to the cerebellum; that the deep roots have particular connections with the peculiar tissues of the protuberance, which we cannot trace into the medulla oblongata; that if, as Sir C. Bell wishes, and as it is reasonable enough to suppose, that the nerves derive their functions from the connections of their origin, it is impossible that the trigeminus and the spinal nerves should have the same functions, since their origins are certainly different. Think not, gentlemen, that the observations with which I shall detain you, are suppositions; I have brought with me specimens which demonstrate the arrangement I shall describe, and we find in many authors passages which justify my observations. Thus, Gall, who has traced the deep roots of the fifth pair, describes them as coming from the cineritious substance at the floor of the fourth ventricle, by several divisions. M. H. Cloquet has described, in an analogous manner, the same origin. Rolando has seen and figured several varieties of the superficial roots. Meckel counts three roots of the fifth pair, a large, deep, and two lesser ones. M. Cruveilhier makes two—one superficial, which is soon lost, and another profound.

Besides, no one before Sir C. Bell had perceived in the trigeminus the two orders of roots, which we can there see to this day through the prism of the system, although we may have discovered nothing new. You now see, gentlemen, whether it is true that the fifth pair resembles the spinal nerves.

I come to the objections of M. Bouillaud—— but I do not perceive him at his place. Being present, I would have answered him; absent, I will not make a reply which he cannot hear. I pass, then, to an examination of the properties of the nerves.

I have already shown how the system of the sensitive and motor nerves, which appears to go back to the school of Alexandria, was developed by Galen, and taught even to the seventeenth century by Dulaurens in his Anatomy; how it was supposed by the ancients to explain the paralysis of sensibility and movement, when isolated or separated from each other; and how Sir C. Bell has revived it for the same end. But I am ready to grant that he has given it an entirely new basis, and that he has made it so brilliant and seductive, that we regret in truth, that he has not been called to the counsels of the Creator.

It is not that this system is false in all its details; on the contrary, we find in it dazzling truths, which reflect great honour on Sir Charles Bell. But what does this prove for the truth of the whole? What system is there that does not contain truths, and even remarkable truths? Were it a single one, it would entice no one, nor would it endure for a day, or rather, it would never become established. Do not wonder, then, that that of Sir Charles Bell has attracted the admiration of my honourable opponents! neither wonder if I combat it as a whole, every where recognising in its details extremely remarkable truths. Be persuaded that my antagonists themselves think in part with me; it is in vain they exalt the system, and declare themselves its defenders; they adopt only that which suits them, and there are not more zealous sectarists than those who think themselves Christians because they go to mass once a year.

To judge of the system, we must then penetrate absolutely into its details. According to Sir Charles Bell, the spinal marrow is composed of three columns or bandelettes on each side of the mesian line, an anterior column destined to motion, a posterior column for sensation, a lateral and intermediate one for the involuntary movements of respiration, etc. The nerves are simple or compound: the simple are those whose roots arise from one and the same part, and have but one property; the compound, those which come from different parts, as from the anterior and posterior columns of the spinal marrow, and have several distinct properties. Consequently, the olfactory, the optic, are simple nerves, and they are endowed with a specific sensibility. Observe, however, that the olfactory arises from three very different parts, by three very dissimilar roots, each of which, besides, does not touch the spinal cord, for the corpus callosum bends itself from above downwards between the roots of this nerve, to embrace within its concavity the superior extremities of the diverging fibres of the corpora striata, as I have demonstrated in my researches on the brain.

As to the optic nerve, it arises by two roots at least, from two parts still very different: the optic couches and tubercula quadrigemina, which have properties very distinct, with the consent of all experimenters. This nerve, so simple from its known properties, is not then so simple from its connections.

The motores oculorum, which all allow to be nerves of motion, do not take their origin precisely from the anterior column of the medulla oblongata, or from the corpora pyramidalia prolonged into the crura cerebri; but principally from a gray matter, and a peculiar yellow substance, which is there found more deeply situated. I have here an old diagram of this description, which I made many years ago, whilst I was engaged in anatomical researches on the nervous system. The drawing not having been made for the occasion, as the antiquity of the paper and the other designs accompanying it will testify, you will not suspect my veracity in this respect. The origin of the third pair of nerves is, then, very different from those roots of the spinal nerves called motor.

The pathetic is, according to Bell, an involuntary muscular nerve, which should come from the lateral column, but which, in truth, comes from the prolongations of the cerebellum into the tubercula quadrigemina and the valve of Vieussiens.

I have not again to point out to you the inaccuracy of the system, or the origin of the fifth pair. It is a task which I have accomplished, in answering the objections of my worthy colleague, M. Blandin. I will not even continue this critical review, through fear of fatiguing the Academy by too many anatomical details. Besides, it suffices, imperfect as it is, to show how a systematic writer bends facts to suit the frame in which he wishes to place them, and how, moreover, it is as much easier for him to find an ideal, in the place of real nature, as he has more intelligence and genius.

My opponents and myself have so often spoken of the experiments of Bell on the fifth pair and the facial nerve, that I will not revert to them. I will merely remark to M. Blandin, that since he supposes that the facial nerve is sensitive at its emergence from the stylo-mastoid foramen, it does not suffice to affirm that it owes its sensibility to other nerves, which join themselves to it in the aqueduct of Fallopius; it must be demonstrated. Until then, his assertion will be only an hypothesis.

It is true, that my antagonists still argue, that as the section of the fifth pair upon the petrous bones paralyzes all the face, and the little sensibility which the facial possesses, it is necessary to conclude that it is not sensitive in itself. Granting this fact to be correct, for which I do not answer, I will say, every one knows that the olfactory, the optic, and auditory nerves, serve for smell, sight, and hearing. If, notwithstanding, the section of the fifth pair on the petrous bone causes the loss of smell, of sight, and of hearing, as M. Magendie teaches, does it prove that these nerves do not serve for smell, sight, and hearing? Does not this fact prove, on the contrary, that the trifacial is only a condition necessary to the functions of these nerves? If, then, the section of the same nerve paralyzes also the sensibility of the facial, how would that prove that the facial is not sensitive in itself? Would it not be more probable that its integrity would be necessary to the sensibility of the facial, as it is to the sensibility of the olfactory, optic, and auditory nerves?

Not being able to examine the system of Charles Bell in all its details, I pass now to the labours of M. Magendie, and shall show you how my opponents, who believe at once in the doctrine of the English physiologist, and in the results obtained by the French physiologist, believe at the same time in two contradictory doctrines. They may, perhaps, with our worthy colleague, M. Bouillaud, reproach me for arraying facts in *civil war, whilst they endeavour, by all their efforts, to reconcile them for the interest of science.* Ah, sirs! does not this frank avowal sufficiently clearly unveil to you that unhappy systematic spirit, against which I have just inveighed? What! shall we have in science, as in politics, ministers of reconciliation! In the sciences, gentlemen, assertions are true or false, and truth does not lay between, and we make not truth by mingling the true and the false! But let us prove, by some examples, how far the experiments of M. Magendie are sometimes contrary to the ideas of Charles Bell. Whilst the latter sees in the fifth pair only, a nerve of motion and sensation, the former shows that its section on the petrous bone, exercises an immense influence on the senses already pointed out above, and without which the functions of smell, sight, and hearing, could not be fulfilled; without which, even the eye cannot preserve its natural integrity, but becoming inflamed, ulcerates, and permits the discharge of the humours. Who would have been able to predict such results? No one. Sir C. Bell, also, whose doctrine is but a system, has not suspected it; he has seen there only a spinal nerve. But will they say, that if MM. Bell and Magendie are not agreed upon the functions of the trifacial, they are upon those of the roots of the spinal nerves. This is nearer the truth, but can this well hold when there has been a common interest in the question. Sir C. Bell having imagined that the spinal nerves ought to receive different properties from each of the two orders of theirroots, bethought himself to irritate, separately, the anterior and posterior roots on a rabbit, to which he assumes not to have caused suffering. Having seen that an irritation of the posterior roots caused no movement in the muscles, whilst that of the anterior did, he concludes from this, on the difference in their functions. But he went no further. M. Magendie, struck with the want of precision in the results of Sir Charles Bell, launched into this new field of experiment, which opened itself before him, and with that ability which characterizes him, lending the aid of his hands to the adventurous conceptions of the London physiologist, he became the arm of the system of which Sir Charles Bell was the head. Behold how, beyond doubt, the interest of that which they regard as their common glory, holds them united and agreed.

It would occupy too much time to relate, in detail, all the discrepancies which exist between the preceding authors, Herbert Mayor, M. Fora, Bellingeri, M. Calmeil, and, among others, myself, who have all repeated and varied the greater portion of the experiments made on the nervous system, to find out their properties.

After having shown, at large, how little agreement there is between the results obtained by different experimenters, let us see if those which pathology furnishes are more favourable to the new doctrine.

The study of diseases shows that the distension and compression of the medulla spinalis, abolishes the manifestation of sensibility and voluntary contractibility of the parts which receive their nerves from a point of the spinal marrow, situated below the lesion, and which I shall call the parts inferior to the lesion. It shows that the disorganization, and especially a solution of continuity of the spinal marrow, abolishes, *ordinarily*, the manifestation of sensibility and of movement in the parts inferior to the lesion. It teaches us that paralysis shows itself generally on the injured side of the spinal cord; that paralysis of sensibility and motion can happen separately or together, without any materially appreciable lesion. These assertions have no need of proof, since their truth is generally, and well known. It is not the case with the following: I shall therefore be obliged to adduce proofs suited to the support of each.

*Sometimes the paralysis manifests itself above a material lesion of the medulla spinalis.* Thus—1. Portal reports to have seen the paralysis of the left arm, and at death a softening, with redness, existing only on the right inferior extremity of the cord, from the last dorsal to the first lumbar vertebræ.

2. A young soldier, who died with universal paralysis, presented only a softening at the lower portion of the cord.

*In certain cases, the lesions of the spinal marrow cause only a paralysis of motion, whatever may be their seat around the axis of the cord.*

3. Preval, who remained in bed during seven years, with the arms and legs flexed and contracted, and which he at times moved voluntarily, presented, at his autopsy, a softening of the anterior columns of the cord, in almost their whole thickness, in their entire length, even to the cerebrum. Why were not the superior extremities, as well as the lower, paralysed, since the alterations of the cord were the same above and below?

4. Elie had a paralysis of motion in his inferior extremities, *only*, after a fall from a third story. According to the new ideas, you would have expected to find, at his death, an alteration of the anterior columns of the cord. A mistake: the spinal marrow was equally softened, both before and behind, on a level with the twelfth dorsal vertebræ.

5. A water-carrier, injured in the back, experienced a complete paralysis of motion in the inferior extremities, and an incomplete paralysis of sensibility. You would diagnosticate, a serious lesion of the anterior columns of the cords below, and a slight alteration of the posterior columns. Still a mistake: the spinal cord was crushed and softened in its whole thickness, and for the extent of an inch opposite the twelfth dorsal vertebra.

6. Mettral died with a complete paralysis of motion in the inferior extremities, and incomplete in the superior. A zealous adherent to the system is not embarrassed; he pronounces boldly: alterations of anterior columns low down the cord, slight in the cervical region; then comes in its turn the autopsy, which shows a semi-fluid softening of the entire lumbar enlargement, although there was no paralysis of sensibility below, and, in short, an induration of the entire cord from the eighth dorsal vertebra to the crura cerebri. How happens it that there was not at last a slight paralysis of the superior members, as well as any thing else ?

7. A child of 7 years died with the signs of a chronic arachnitis, and there were found encephaloid masses in the inferior and posterior part of the cerebellum which compressed the cord above and behind. The restiforme and olivary bodies were altered and reduced in encephaloid matter. There was no paralysis of any sort; not the slightest trouble in comparison with a lesion so grave as that of the cerebellum! What disobedience to the laws so often proclaimed here by M. Bouillaud! What impudence on the part of this child.

Why was the sensibility alone preserved in the 4th, 5th, 6th, and 7th cases of which I have spoken? Is it that generally it is less easily paralysed than motility? It is at least what I have gathered from the mass of observations which I have united and reflected on.

*But pathology shows still more astonishing discrepancies than those with which I have occupied your attention.* 8. Petitpas died with a strangulation of the spinal cord, caused by enlargement of the posterior portion of the second vertebra, with the first softening without disorganization, situated behind the cord; with a second softening of an analogous kind extending from the sixth cervical to the second dorsal vertebra; lastly, with a third softening extending from the seventh dorsal vertebra to the lumbar enlargement, and reduced into a semi-fluid subtance below. These were the lesions: I defy all physiologists and pathologists to indicate now the attending phenomena which were the consequences of them. Here they are: the neck and the chest were benumbed to the third rib, from above downwards; the superior extremities, and the trunk, from below upwards to the epigastrium, were also benumbed, and were incessantly tormented by intolerable itchings. Between the third and seventh ribs, the sensibility was undiminished around the chest. Why were the upper lesions of the cord sufficient to alter the sensibility of the superior parts, and were not sufficient to alter that of the parts placed below, between the third and seventh ribs?

9. Delafoix died with a cerebro-spinal congestion, with remarkable softening of the cervical enlargement for the extent of two inches, particularly of the gray substance. These lesions being given, who would venture to guess the symptoms which were the consequence of them? He had paralysis of the superior extremities, and why not of the inferior?

10. Dufont died with softening and disorganization of the spinal marrow, on a level with the processus dentata, which was moveable; with a rupture of the medulla oblongata, probably made after death; a rupture of the motor externus, pneumo-gastric, and glosso-pharyngeal nerves, with alteration of the facial, auditory, and hypoglossal nerves. He who would venture to guess the symptoms produced by these lesions, would be quite sure to be deceived! There were severe, superficial, and deep-seated pains in the neck, twelve hours only before death; imperfect paralysis of the muscles which elevate the lower jaw; and lastly, prostration, or a sort of general paralysis.

11. M. L—— died with a paralysis of motion of the superior extremities, but without any paralysis of the inferior. What would you assign as the lesions of the cord? It was liquid, fluctuating in a membranaceous tube between the fourth cervical and the fourth dorsal vertebra. It presented on the left, for half an inch in extent, lenticular portions, and a bandelette, altered for two lines in breadth, which appeared to M. Magendie so incapable of transmitting sensations and volition, that he asked whether the membranes of the spinal cord might not be the agents of this transmission.

*Lastly. Pathology sometimes shows sensation and motion preserved in parts entirely cut off from the brain, in consequence of the absence, destruction, and solution of continuity of the spinal marrow in its whole thickness.* You ought, gentlemen, to be prepared for this proposition by the facts cited above, and particularly by the eleventh.

12. Famel showed, in 1711, at the Académie des Sciences, an infant at term, without cerebrum, cerebellum, or spinal marrow, who lived two hours, and felt the baptismal water at the moment that it was christened. (And how do you know it? cried a member of the Academy.) As one knows that a leech feels the pepper that is thrown on its tail, when we see it agitated and doubling itself on itself. For every movement consequent upon a tcuch is not a communicated or a mechanical movement; it is the result of an impression, and, consequently, of a sensation. Thus, when we touch an actinite spread out in the waters of the sea, and which closes itself suddenly—when we touch a polypus, and it moves—when we prick the sensitive plant, without shaking it, or when we cauterize it with an acid, its leaflets, its leaves, its branches even, close themselves, every one says that these beings feel, and that they have sensibility, because they have expressed it by their movements. It is thus by its movements that the child in question evinced that it had felt. And why should it not execute movements, when an acephalous fœtus executes them in its mother's womb,—when a fly flies with its head cut off,—when a salamander swims, deprived almost entirely of the encephalon and of the head,—when a duck runs several steps, its head being taken off?

13. Mery saw, in 1712, an infant, who lived twenty-one hours, and took some nourishment, although he had neither brain nor spinal marrow. As he took the nourishment, he must have felt the need of it,—and as he accepted it, he must have felt that it was presented to him, and have made movements necessary to seize and swallow it.

14. Rippert, a Marseillais, wounded on 10th August by a ball which passed through the lungs and the tenth dorsal vertebræ, urinated, moved the pelvis without convulsions, and the inferior extremities, until his death. At his autopsy, the spinal marrow was found cut across between the tenth and eleventh dorsal vertebræ. Desault perceived so well the extraordinary nature of this fact, that the pupil who reported the observation over his name in his journal, presents it as a case which subverts all received ideas. Thus has it been examined with the greatest attention by a man eminently capable of properly observing a fact of this nature.

15. In 1820, a scrofulous child died without any paralysis, either of sensation or motion, and notwithstanding the autopsy showed a complete interruption of the spinal marrow from the ninth dorsal to the first lumbar vertebra.

16. Chardonnens died with an equally inappreciable lesion, and yet the inferior half of the lumbar enlargement was found softened and destroyed, even to three or four lines above the section. The greater part of the nerves of the pelvic extremities were detached from their origin, and the others adhered only by the dura mater.

17. A man of 30 years died without any evident paralysis: at the autopsy no trace of the cord from the tenth dorsal vertebra to its lower extremity; its substance was different, nervous trunks visible only in the foramina of their junction.

18. A man named Dubois died in 8 days of a grave fever, with cramps in his legs, and after having gotten up, himself, to go to stool, a few hours before his death. At the autopsy, the spinal cord was interrupted for the extent of an inch and a half, about the sixth dorsal vertebra, with an intermediate yellowish liquid.

Here are eighteen cases which do not agree, either with the new, or even with our ancient ideas on the functions of the nervous system. But the seven, and I might say the eight last especially, are well calculated to excite astonishment. Moreover, when we bring to mind that the most of these facts, all of the gravest nature, date within late years; that they are becoming more numerous, because now autopsies are made more exact than formerly; that besides, they come to us from well known and estimable authors, for I have selected them with the utmost care, we can scarce refuse to admit that were we oftener to open the vertebral column, we should be in possession of a greater number, and that probably they will multiply themselves still more, in proportion as we shall further study the diseases of the spinal marrow.

